# Can Predicted Protein 3D Structures Provide Reliable Insights into whether Missense Variants Are Disease Associated?

**DOI:** 10.1016/j.jmb.2019.04.009

**Published:** 2019-05-17

**Authors:** Sirawit Ittisoponpisan, Suhail A. Islam, Tarun Khanna, Eman Alhuzimi, Alessia David, Michael J.E. Sternberg

**Affiliations:** Structural Bioinformatics Group, Centre for Integrative Systems Biology and Bioinformatics, Department of Life Sciences, Sir Ernst Chain Building, Imperial College London, London SW7 2AZ, UK

**Keywords:** PDB, protein data bank, SIFT, Sorting Intolerant From Tolerant, missense variants, structure-based prediction, protein structure prediction, variant effect prediction, Phyre2 protein structure prediction

## Abstract

Knowledge of protein structure can be used to predict the phenotypic consequence of a missense variant. Since structural coverage of the human proteome can be roughly tripled to over 50% of the residues if homology-predicted structures are included in addition to experimentally determined coordinates, it is important to assess the reliability of using predicted models when analyzing missense variants. Accordingly, we assess whether a missense variant is structurally damaging by using experimental and predicted structures. We considered 606 experimental structures and show that 40% of the 1965 disease-associated missense variants analyzed have a structurally damaging change in the mutant structure. Only 11% of the 2134 neutral variants are structurally damaging. Importantly, similar results are obtained when 1052 structures predicted using Phyre2 algorithm were used, even when the model shares low (< 40%) sequence identity to the template. Thus, structure-based analysis of the effects of missense variants can be effectively applied to homology models. Our in-house pipeline, Missense3D, for structurally assessing missense variants was made available at http://www.sbg.bio.ic.ac.uk/~missense3d

## Highlights

• Interpretation of genotype–phenotype relationship is a major challenge in modern genetics.
• Structural analysis can differentiate neutral *versus* pathogenic missense variants.
• Three-dimensional protein models are as effective as experimental structures to infer the pathogenic effect of a variant.
• Structural analysis should be routinely implemented in the pipeline for variant effect prediction.
• A server Missense3D is available to the community to perform the structural analysis on uploaded coordinates and variants.

## Graphical abstract

**Figure:**
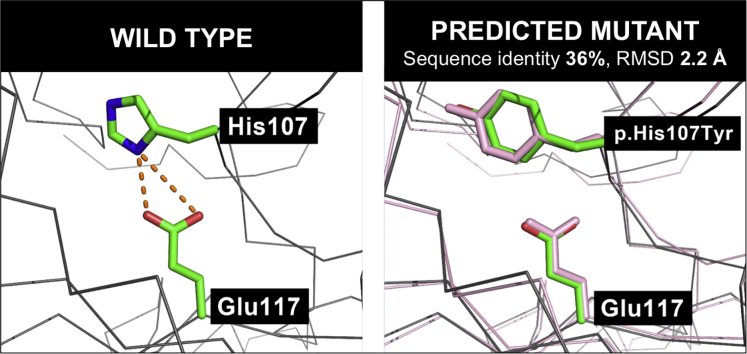

## Introduction

One the major challenges in modern genetics is predicting the effect of the overwhelming number of variants being revealed through sequencing projects. This is particularly important in analyzing variants occurring in the human population that could be involved in the pathogenesis of disease [1], [2]. It has been noted [3] that in many studies the challenges and costs arise more from the analysis of the data than the actual sequencing. To help prioritize potentially deleterious variants, groups have developed *in silico* prediction programs. Many of these methods rely primarily or exclusively on sequence-based features, such as amino-acid evolutionary conservation, for example, Sorting Intolerant From Tolerant (SIFT) [4], FATHMM [5], MutationTaster [6] and Condel [7]. Although many of these approaches provide a high sensitivity (i.e., a high true-positive rate, or TPR), they often also have a low specificity [8] (i.e., a high false positive rate, or FPR). REVEL [9], an ensemble method that combines features from several commonly used predictors, showed the best discriminatory ability to distinguish between damaging and neutral.

A major limitation of *in silico* predictors is that they often return a binary outcome (i.e., neutral or deleterious) and provide little or no explanation of the effect of the substitution. In many applications, including clinical genetics [1], [2], a high specificity coupled with an explanation of the phenotypic effect is of major benefit in prioritizing further studies.

Since the structure of a protein is intimately linked to its stability, function and interactions, many *in silico* prediction methods employ knowledge of protein structure, either exclusively or in combination with sequence-based features, with the aim of providing high-quality predictions (high TPR and low FPR). Although the coordinates in the protein data bank (PDB) [10], [11] cover only about 17% of the residues of the human proteome, this coverage can be markedly extended to a total of about 50% by protein structure prediction [12]. Our recent (unpublished) analysis using an updated version of Phyre2 yields a coverage by confidently predicted models of 54% of the residues of the human proteome. Local features, such as the regular secondary structures, residue burial, transmembrane-spanning regions and disorder, can be predicted proteome-wide, for example, Ref. [13]. Models for tertiary 3D structure can be predicted from the known coordinates of one or more structural templates, and this approach is commonly called homology, comparative or template-based modeling.

There are a variety of strategies to prioritize potentially damaging amino acid substitutions that benefit from the additional information that can be obtained from protein structure, with some approaches only applicable when there is a PDB structure and others that include information from structure prediction, either of just local features or of a 3D model or both (reviewed in Ref. [14]). Approaches that predict local structural features but not tertiary structure include SNAP2 [15] and our algorithm SuSPect [16] and the widely used PolyPhen2 [17]. Other methods assess the free-energy change resulting from the amino-acid substitution with algorithms using either explicit potential energy functions (e.g., FoldX [18]) or employing knowledge-based or machine-learning derived parameters, for example, PopMusic [19], mCMS [20] and DUET [21]. Some approaches closely integrate sequence and structural analysis, for example, INPS-MD [22]. The machine-learning approach ENTERPRISE [23] uses the coordinates of predicted protein structures to assess the effect of the substitution on residue–residue interactions.

A major limitation of these structure-based approaches is that they provide little or no insight into the mechanism by which an amino-acid substitution affects protein structure. There have been several studies [24], [25], [26], [27], [28], [29], [30] analyzing the structural effects of missense substitutions. It is well established that missense variants in the core of the tertiary structure are more often associated with disease than those on the surface. In addition, analyses (see Ref. [31], including from our group [32], [33], have shown that variants at protein/protein interfaces are enriched in disease-associated missense variants compared to those on the remainder of the surface.

Given the additional insight provided by a stereochemical description of the effect of a missense variant, computational methods such as SNPs3D [26], [27] and Hope server [34] have been developed to report these effects. However, the inability to upload a 3D coordinate file and the extensive time required to return results are major limitations. SDM2 [35] predicts changes in protein stability upon amino acid substitution by uploading a 3D coordinate file (PDB or model) and provides a report of the stereochemical effect. A related approach Single Amino Acid Polymorphism pipeline (SAAPdap) [36] reports features such as breakage of H-bonds, burying a charge and introducing a clash. However, the user cannot upload his/her own set of coordinates such as would be obtained via homology modeling.

Many of the structure-based approaches, particularly those estimating free energy changes, have been benchmarked solely on experimental structures, while in others, experimental and predicted structures are pooled in the evaluation. Sometimes, web servers can only be used to analyze a PDB coordinate file. The problem is that when using a predicted structure, one should ask: are the predictions obtained using a 3D model similar to those that would be obtained using an experimental structure? And if so, to what extent does the accuracy of models affect these results?

To address this, we have compared the stereochemical effects resulting from a missense variant using PDB coordinates and homology-predicted structures generated over a range of sequence identities between query and template. We show that similar TPRs and FPRs are obtained when using homology models compared to the results on PDB structures. Our in-house pipeline used for this analysis and named Missense3D is available at http://www.sbg.bio.ic.ac.uk/~missense3d.

## Results

### Overview of variants structural analysis and its evaluation

**Fig. 1:**
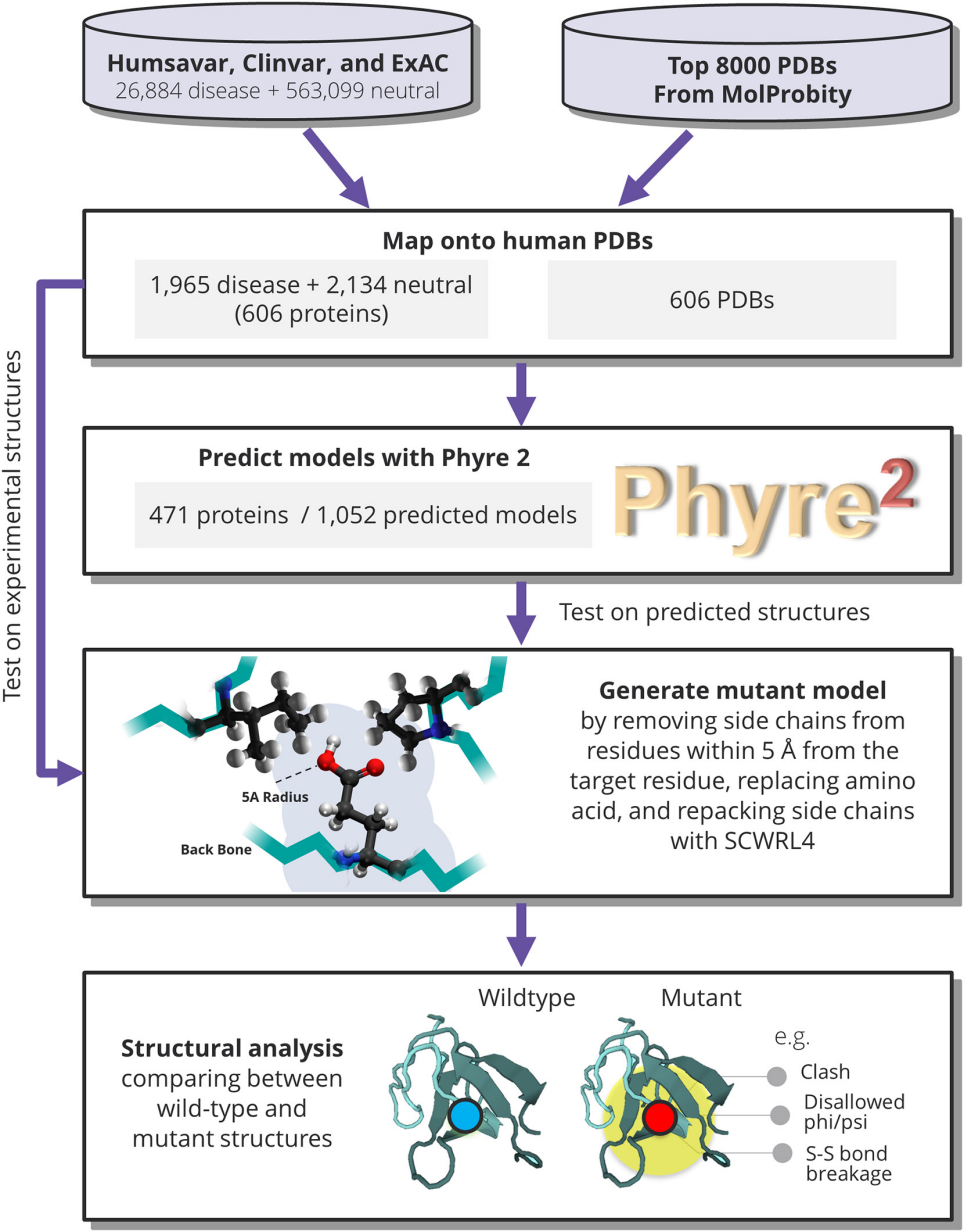
Pipeline to analyze the structural impact of missense variants in experimental and predicted structures.

Our data consist of 606 human protein structures, which were obtained from the MolProbity [37] top8000 database of high-quality coordinates. Onto these structures, we mapped 1965 disease-associated missense variants and 2134 missense variants with no known disease association reported to-date (which we will from now on refer to as “neutral” variants) obtained from Humsavar [38], ClinVar [39], and ExAC [40] and occurring in 606 human proteins (the pipeline for this analysis is presented in Fig. 1).

The mutant structure (MUTANT) was generated from the wild-type coordinates using SCWRL4 [41]. The side chain of the target residue (residue to be substituted) and the side chains of any residue within 5 Å from the target residue (defined by any pair of inter-residue atoms closer than 5 Å) were removed from the coordinates. The side chain of the target residue was replaced with the mutant side chain, the wild-type side chains of the neighboring residues reintroduced and then repacked using SCWRL4 to generate the coordinates of the MUTANT. Since SCWRL4 can slightly re-adjust all side chains even if they are labeled as fixed, the input wild-type 3D coordinates in which each side chain was specified as fixed but still subjected to minor adjustment by SCWRL4 were also generated (WT). SCWRL4 does not readjust the backbone.

The MUTANT and WT structures were analyzed to identify whether the structural consequence of the substitution is expected to be damaging in terms of the stability of the folded protein. Based on well-established principles of protein conformation and previous studies on the structural consequences of disease-associated substitutions [26], [27], [28], [29], [30], [36], we considered 17 structural features (see Table 1 and Methods). For the three structural features calculated using a distance (disulfide bonds, hydrogen bonds and salt bridges), 1 Å was added to standard distances to allow for errors in the modeling by SCWRL4 and errors in the predicted structure. The aims were to provide an analysis describing structurally damaging changes introduced by human missense variants, which can suggest that a variant is likely to be disease causing, and to compare results obtained using experimental and 3D model structures. We only require a single alert to be identified and we are not combining scores. Missense3D, the algorithm used for this analysis, is available as a webs erver to study the structural consequences of any missense variant from any species.

**Table 1:** Structural features evaluated in this study and used by Missense3D web server

Feature assessed as a structural impact	Description
Disulfide bond breakage	The substitution breaks a disulfide bond that was in the wild-type. The maximum S–S length for the bond is 3.3 Å.
Buried Pro introduced	The substitution introduces a buried proline.
Clash	The mutant structure has a MolProbity clash score ≥ 30 and the increase in clash score is > 18 compared to the wild type.
Buried hydrophilic introduced	The substitution replaces a buried hydrophobic residue with a hydrophilic residue.
Buried charge introduced	The substitution replaces a buried uncharged residue with a charged residue.
Buried charge switch	The substitution switches the charge (+/−) of the buried residue.
Secondary structure altered	The substitution results in a change in the DSSP secondary structure assignment at the variant position.
Buried charge replaced	The substitution replaces a buried charged residue with an uncharged residue.
Disallowed phi/psi	The mutant residue is in an outlier region, while the wild-type residue in both the 3D coordinates input file and the WT are in the favored or allowed regions.
Buried Gly replaced	The substitution replaces a buried glycine.
Buried H-bond breakage	The substitution breaks all side-chain/side-chain H-bond(s) and/or side-chain/main-chain bond(s) formed by the wild-type residue which was buried. The maximum H-bond N–O length is 3.9 Å.
Buried salt bridge breakage	The substitution breaks a salt bridge formed by the wild-type residue which was buried. The maximum N–O bond length is 5.0 Å.
Cavity altered	The substitution leads to an expansion or contraction of the cavity volume of ≥ 70Å^3^. Cavity also refers to a pocket on the surface.
Buried/exposed switch	The substitution results in a change between buried and exposed state of the target residue. (RSA < 9% for buried and the difference between RSA has to be at least 5%.)
Cis Pro replaced	The substitution replaces a proline, which was in cis configuration in the wild type.
Gly in a bend	The substitution replaces a glycine, which is located in a bend curvature (reported “S” in DSSP).
Exposed hydrophobic introduced (evaluated but not used)	The substitution replaces an exposed hydrophilic residue with a hydrophobic residue (not employed as a feature in Missense3D).

### Results on experimental structures

A disease-associated variant identified as having a damaging structural impact is regarded as a true positive (TP) since the features are designed to identify a major disruption to the folded structure. However, since a missense variant may cause disease by affecting features, which are currently not considered in our analysis, such as a residue critical for function or ligand binding, our TPR could be enhanced by a further consideration of such features. We consider a neutral missense variant identified as being structurally damaging by our analysis as a false positive (FP). However, this is likely to overestimate the FPR since damaging a protein structurally may not result in a disease if the corresponding gene is non-essential or haplosufficient. Moreover, some missense variants may actually be disease associated, but this has not yet been identified.

**Fig. 2:**
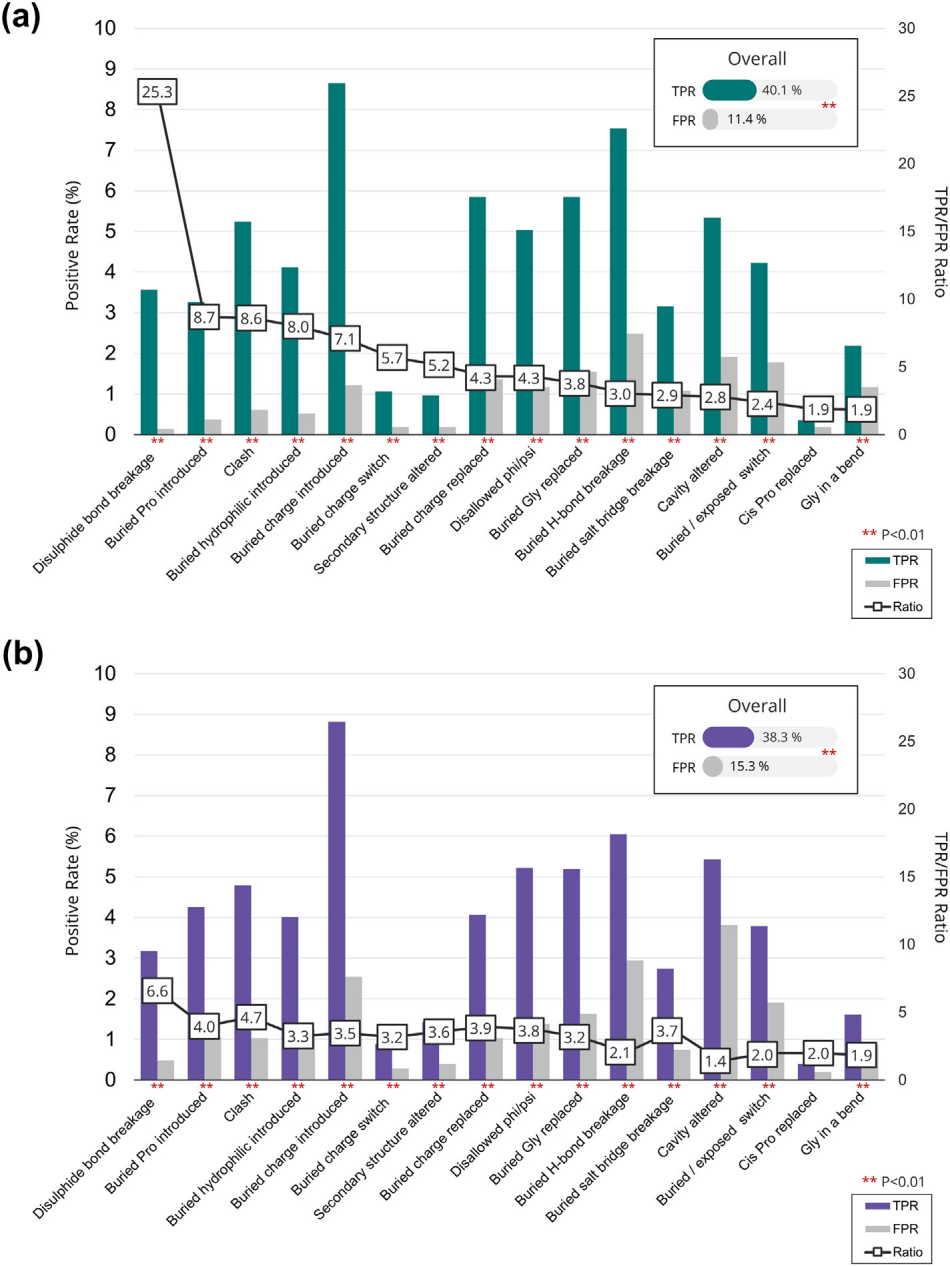
Performance of structural analysis on experimental structures. For each feature, the TPR on disease-associated (Disease) and the FPR on neutral (Neutral) variants are plotted as bars. The ratios of the true-positive to false-positive rates (TPR/FPR) are given, and for ease of viewing, these are connected by a line. The overall TPR and FPR on the entire data set are also reported. Significance at *P* < 0.01 (denoted by **) is evaluated in a one-tailed test of the difference of two proportions. Panel a are the results from the set of high-quality X-ray structures (resolution < 2.0 Å) from MolProbity's Top8000 database [37]. Panel b are the results on a second independent data set of 855 structures with lower resolution (< 2.5 Å) from the PDB.

Overall, a structural analysis is able to distinguish disease-associated from neutral variants. In particular, Fig. 2a shows the performance of each of the 17 structural features assessed to distinguish disease-associated and neutral variants based on the fraction of their rates (TPR/FPR) (see also Table S1). The fraction is greater than 1.0 for 16 of the 17 features. Of these 16, for 15 features, the TPRs and FPRs are significantly different (*P* < 0.01). These differences remain significant (*P* < 0.01) after Benjamini–Hochberg [42] correction for 17 multiple tests. For completeness, we have added the results that would be obtained by using the default cutoff distances for disulfide bonds, salt bridges and H bond in Table S2. Although TPR/FPR ratio for each feature remained similar, the results obtained by using the relaxed cutoffs allow explaining the effect of more variants.

“Cis Pro replaced” is not significant (*P* = 0.3) despite a TPR/FPR ratio of 1.9 because there are few observations, which limits the power of the test—proline cis peptides are not common in proteins [43] and there were only seven disease-associated and four neutral variants in our data set. However, as it is well established that non-proline cis peptides are exceptionally rare in proteins [43], this feature was retained in the list of structural alerts. The feature “exposed hydrophobic introduced” proved not to be effective in identifying structural alerts for disease variants with a TPR/FPR ratio of 0.6. This result is consistent with the observation that a large part of the protein surface is formed from hydrophobic residues [24], [44].

Breaking a disulfide bond was the most discriminating structural alert with a TPR/FPR ratio of 25.3. There were only three variants classified as neutral where a wild-type disulfide bridge was identified as broken. These variants were in C-type lectin domain family 4 member (*CLC4A*, UniProt ID:Q9H2X3, p.Cys381Arg, residue Cys 381 in PDB ID:1XPH), beta-2 glycoprotein 1 (*APOH*, UniProt ID:P02749, p.Cys325Gly, Cys 306 in PDB ID:3OP8) and E-selectin (*SELE*, UniProt ID: P16581, p.Cys130Trp, Cys 109 in PDB ID:1G1T). These are all structures from the high-quality MolProbity top 8000 database, and manual inspection confirmed a disulfide bond in the wild type. One expects breaking a disulfide bond will markedly reduce protein stability, and hence, the variant should be disease associated. One possibility is that the assignment of these variants as neural is incorrect, and Missense3D correctly identified these as disease associated. Indeed, p.Cys381Arg in *CLC4A* (dbSNP:rs184828145) and p.Cys130Trp in *SELE* (dbSNP:rs5360) are extremely rare variants (ExAC allele frequency of 0.00007414 and unknown, respectively). Moreover, p.Cys325Gly in *APOH* (dbSNP:rs1801689), although reported as “polymorphism” in UniProt, has been shown to affect APOH binding to phosphatidylserine when present in compound heterozygous patients, and it has been suggested to impair APOH binding to phospholipid in homozygous patients [45]. Thus, the breakage of the disulfide bond identified by our structural analysis could suggest that these variants may have an impact on these proteins.

The next most discriminating feature was introducing a proline in a buried residue. The allowed backbone phi and psi torsion angles are substantially reduced for proline compared to any other residue, and thus, introducing a proline can disrupt the structure [46]. In a buried location, it is harder for the backbone to rearrange to accommodate the introduction of a proline. Four of the next five most discriminating features relate to the introduction or alteration of a hydrophilic or charged side chain into the protein core, consistent with the principle that an unpaired charged or polar group in the core nearly always destabilize the protein [47].

As the backbone is not altered by SCWRL4, changes to the secondary structure as defined by DSSP [48] can only result from the introduction or removal of a proline, which alters the backbone hydrogen-bonding pattern. There were 22 disease-associated (1.12%) and 5 neutral substitutions (0.23%) that altered the secondary structure determined by DSSP.

One might expect that replacing a buried glycine with any residue would generate clashes. However, we found that not all buried glycine substitutions resulted in clashes: 87 out of the 115 disease-associated variants (75.7%) and 30 out of the 33 neutral variants (90.9%) did not result in high clash scores.

The TPR values in Fig. 2a indicate the relative frequencies of observing particular damaging structural effects in the disease-associated variants. Our results are broadly in agreement with analyses by others [24], [26], [27], [28], [29], [30], [36]. In our data set, the most common disruptive feature is introducing a buried charge, with 8.7% of disease-associated variants affected. The other common effects (TPR > 5%) are as follows: breakage of a buried H-bond, replacing a buried charge, replacing a buried glycine, introducing a disallowed phi/psi, altering a cavity, and introducing a clash. In addition, introducing a hydrophobic residue onto the surface has a TPR of over 5, but this feature occurred more often in neutral variants.

Overall, 40.1% (788) of the disease-associated and 11.4% (244) of the neutral variants were identified as having at least 1 of the 16 structural damaging changes [TPR/FPR 3.51, *P* < 0.01; Matthews Correlation Coefficient (MCC) = 0.33]. We compared these results with those obtained using mutant structures generated using FoldX [18]. Although the overall TPR remained the same at 40.0%, the FPR for FoldX-generated mutants was higher (16.0%) yielding a poorer TPR/FPR of 2.5 (*P* < 0.001, McNemar test; Tables S3–S5). Overall, 91% of variants (3749/4099) were predicted to have a similar effect regardless of the tool used (SCWRL4 or FoldX). In particular, at disease variant level, the predictions were identical in 70% (636 variants), different in 3% (25 variants) and similar (in cases were multiple features were triggered, at least one feature was the same in both mutants) in 27% (242 variants). When results for individual features were analyzed (Table S6), the TPRs and TPR/FPR ratios remained similar for all features with the exception of clash, which was lower for FoldX generated mutants. This difference may be explained by the fact that this parameter is mutant structure dependent and was developed for SCWRL4-generated structures.

The TPR of the structural analysis was confirmed on an independent data set of 855 structures with lower resolution (< 2.5 Å) corresponding to 565 unique human proteins. A total of 3718 disease-causing and 2516 neutral variants (extracted and classified according to UniProt, Humsavar database) were mapped and structurally analyzed. The TPR on this independent data set was 38% (number of true positives 1424 out of 3718, *P* = 0.18 on a comparison of proportions test; MCC = 0.25). Moreover, we confirmed that for 15 out 16 features analyzed, the TPRs and FPRs were significantly different (Fig. 2b, *P* < 0.01 after Benjamini–Hochberg correction and Table S7). Interestingly, in this data set of 3D structures with a lower resolution, we observed a higher FPR (15%, no. of false positives 386 out of 2516; *P* < 0.01 on a comparison of proportions test), which may be due to the lower quality of the structures analyzed.

### Analysis of rare, common and unknown neutral missense variants

We analyzed the structural consequences of three subsets of neutral variants based on their minor allele frequency (MAF): 273 common (MAF ≥ 0.01), 1550 rare (MAF < 0.01) and 311 unknown (no MAF available). The FPRs for common, rare and unknown neutral variants from the structural analysis were 5.9%, 11.6% and 15.8%, respectively. Interestingly, when using the SIFT algorithm, which assess the deleterious effect of a variant purely by sequence conservation, the FPRs for the same sets were 29.3%, 48.2% and 51.1%. It has been suggested [49], [50] that several rare variants currently assigned as neutral may subsequently prove to be disease associated. Moreover, several variants, which are currently considered of uncertain clinical significance, may be associated with disease [51]. We observe that roughly double the number of rare variants generated structural alerts compared to common variants, suggesting that several rare variants may be damaging. Similarly, several of the variants in the unknown MAF subset that raised a structural alert may be disease associated.

### Results on homology models

**Fig. 3:**
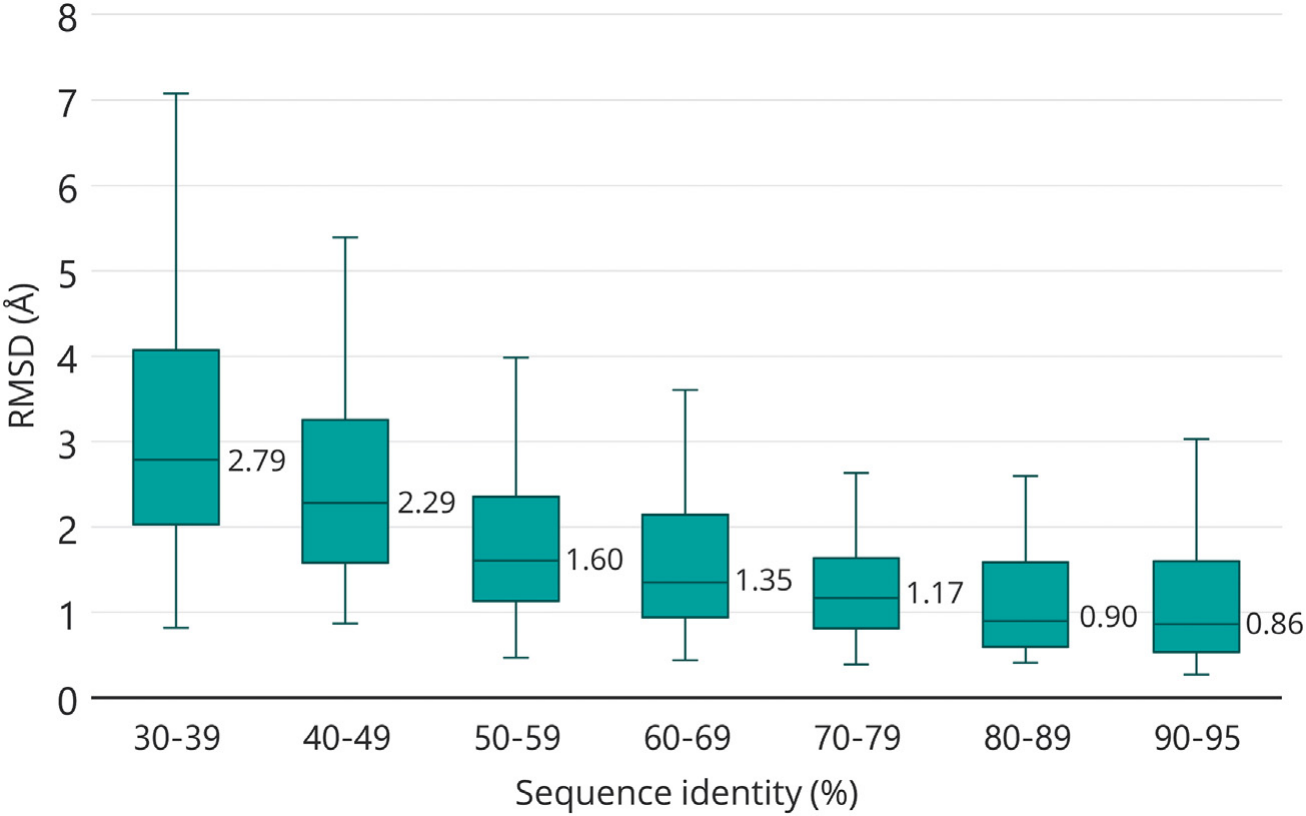
The distribution of the RMSD (Å) between the Phyre2-predicted models and the true experimental coordinates binned according to the % sequence identity between query and template. The central line in a box is the median RMSD with its value reported. The upper and lower box boundaries are the upper and lower quartiles. The whiskers extend up an additional 1.5 × the difference between the median and the upper quartile and down an additional 1.5 × the difference between the median and the lower quartile.

A total of 1052 Phyre2-predicted homology models (median length, 247 residues; range, 52–1183 residues) were obtained for 471 experimental PDB structures over a range of sequence identities between the query and template. Figure 3 shows the distribution of RMSDs of equivalenced Cα atoms between the predicted and corresponding experimental structures. As expected, the median RMSD increases with decreasing sequence identity between query and template. The RMSD median was 0.86 Å for models in the sequence identity range 90%–95% and the median increased to 2.79 Å for the lowest identity range (30%–39%).

**Fig. 4:**
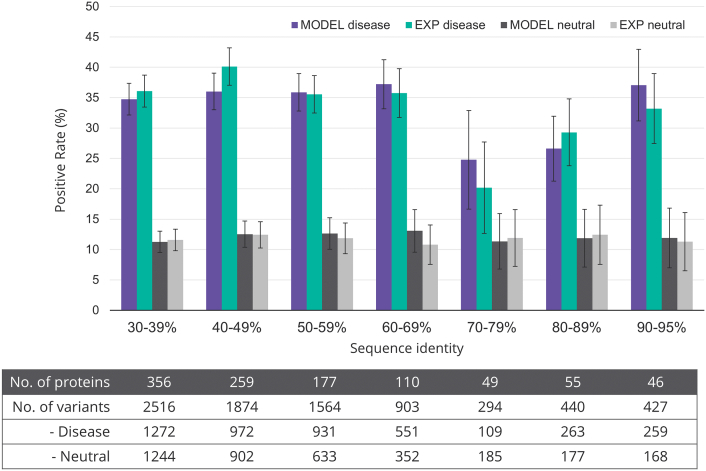
Performance of structural analysis on predicted models at different sequence identities. For each sequence identity bin, the fractions of positive predictions for the disease-associated and neutral variants are shown for both the predicted and the corresponding experimental structures. 95% Confidence intervals on the positive rates are shown as lines.

Figure 4 shows the results of the structural analysis on Phyre2 homology models (see also Table S8). In each sequence identity bin, the results for the models and the experimental structures corresponding to the models in that bin are reported. The relatively low numbers of proteins in the 70% and 80% bins are due to the lack of templates in these sequence ranges leading to far larger confidence intervals for the TPR and FPR, which hinders comparisons. In general, similar TPRs and FPRs are observed for models and experimental structures. Despite some fluctuation in the TPRs at different sequence identities, the percentages of the FPRs in all bins remained broadly stable between 11% and 13%. Moreover, there was on average 88% (range, 86%–92%) agreement at variant level between the predictions obtained from models in different sequence identity bins compared to those obtained from the corresponding experimental structures (Table S9). Since some of the features are dependent on the quality of the model, we re-analyzed the data using only the 10 wild-type dependent features. The results showed that new TPR/FPR ratio obtained using experimental structures remained overall similar to that obtained using models (Table S10). These results show that overall the performance of the structural analysis does not deteriorate substantially even when the structural model is based on a low-sequence identity template. The successful extension of these results from experimental to predicted structures is, in part, the result of using relaxed cutoffs (addition of 1 Å to distance calculation) in the identification of the structural alerts.

Supplementary Figure 1 presents the TPR and FPR for each of the 16 features for the models within each sequence identity bin and compares them to the results for the corresponding experimental structures. We assessed the results on whether the ratios of TPR to FPR for the predicted structures are similar to those for the corresponding experimental structures within that bin. By inspection, the 16 features can be separated into two groups based on whether they still maintain good performance (ratio of TPR/FPR) even when there is a low-sequence identity of the model to template. Features that tend to maintain good performance even at low-sequence identity rely on the buried or exposed state of the wild-type or mutant residue, on the backbone conformation, changes involving glycine or proline, and on the cavity volume. This is because homologous structures, even with low-sequence identity, tend to preserve the backbone conformation more than the side-chain positions [52] and residues tend to maintain their buried or exposed status [48].

In contrast, the performance of the features requiring the measurement of a distance (i.e., the disruption of hydrogen bonds, salt bridges and disulfide bonds) drops when the sequence identity is low largely the consequence of poor modeling of the side-chain position. There is also a drop in the discriminating power of the clash feature coupled with a marked reduction in both the TPR and FPR at low-sequence identity. However, this does not mean that there were fewer clashes in the low-sequence identity homology models. As expected, these models had more clashes than the crystal structures, but our criterion for clash requires a marked difference (> 18) in the scores between the wild-type and the mutant structures.

**Fig. 5:**
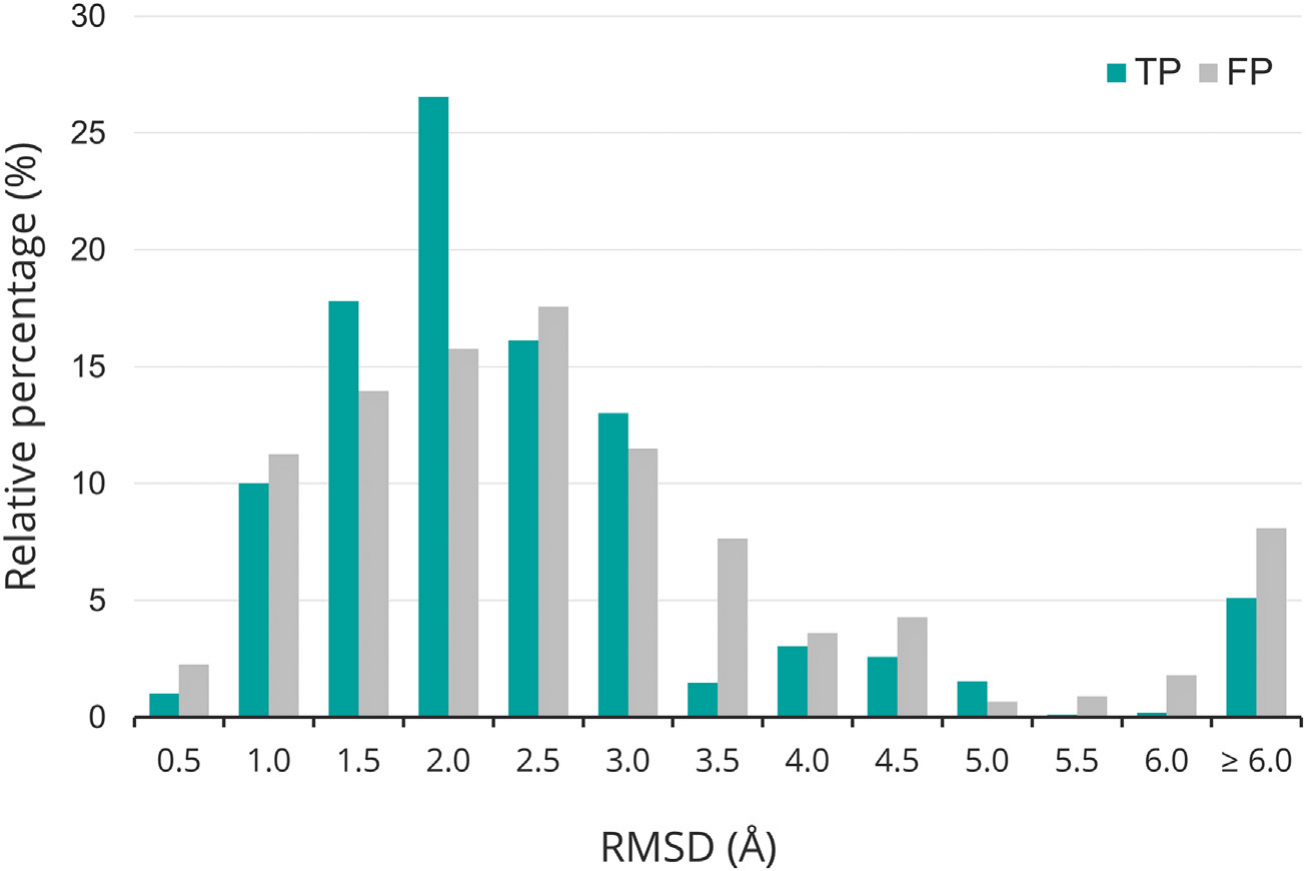
Histogram of the relative frequencies of RMSD (Å) of predicted models grouped according to whether the variant was a TP and FP. The bin labeling shows the upper bound; for example, 0.5 denotes the range 0.0 Å ≤ RMSD < 0.5 Å. The last bin is RMSD ≥ 6 Å. The relative percentage displayed on the *Y* axis is the fraction of true positives and similarly the fraction of false positives in each bin range.

Figure 5 shows the distributions of the relative frequencies of the RMSDs of the predicted models for the TP and FP assignments. A one-tailed Mann–Whitney *U*-test confirms that the distribution of RMSDs for the TP predictions is significantly lower (*P* < 10^−4^) than for the FP predictions (performed on the total frequencies). However, as there remains a large overlap in RMSDs, the overall quality of the model does not provide a reliable guide as to whether the structural analysis will yield a correct identification of a damaging effect.

### Case studies

We present two case studies to illustrate the modeling of the structural impact of missense variants and the consequences of examining predicted models rather than the experimental structures.

**Fig. 6:**
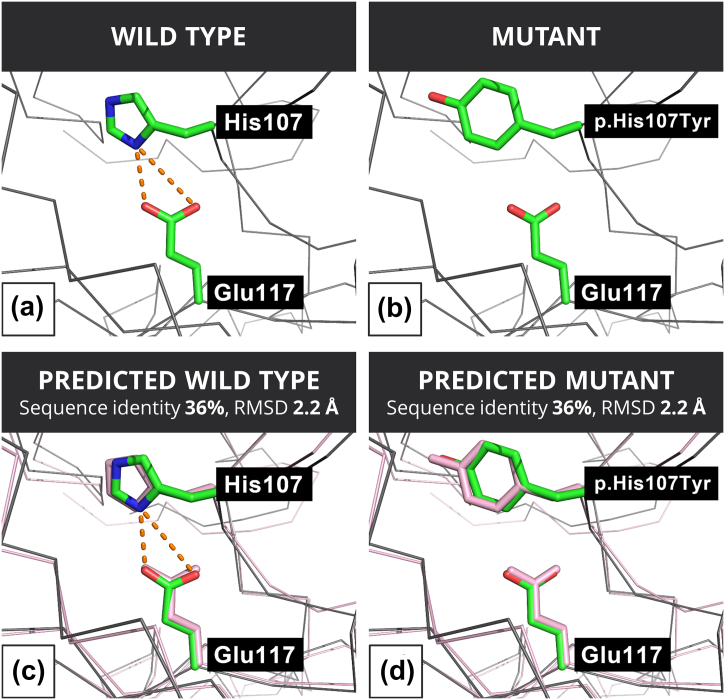
Structural analysis of p.His107Tyr in the experimental and predicted structures of carbonic anhydrase (CA2). (a) His 107 in the wild-type (WT) (PDB ID: 2FOS), (b) Tyr 107 in the mutant (MUTANT) modeled from the WT, (c) Phyre2-predicted structure of the wild type with His 107 (PREDICTED WILD TYPE) and (d) predicted structure of the Tyr 107 mutant based on the Phyre2-predicted wild-type structure (PREDICTED MUTANT). In all four panels, the Cα traces of the structures analyzed by Missense3D are shown in gray. In the predicted structures (c and d), the Cα trace and side-chain positions shown in panels a and b are shown in pink. The side chains of the His 107, Tyr 107 and Glu 117 are show by chemical type (green for non-polar, blue for positive and red for negative). Figures were generated using PyMOL [54].

**Fig. 7:**
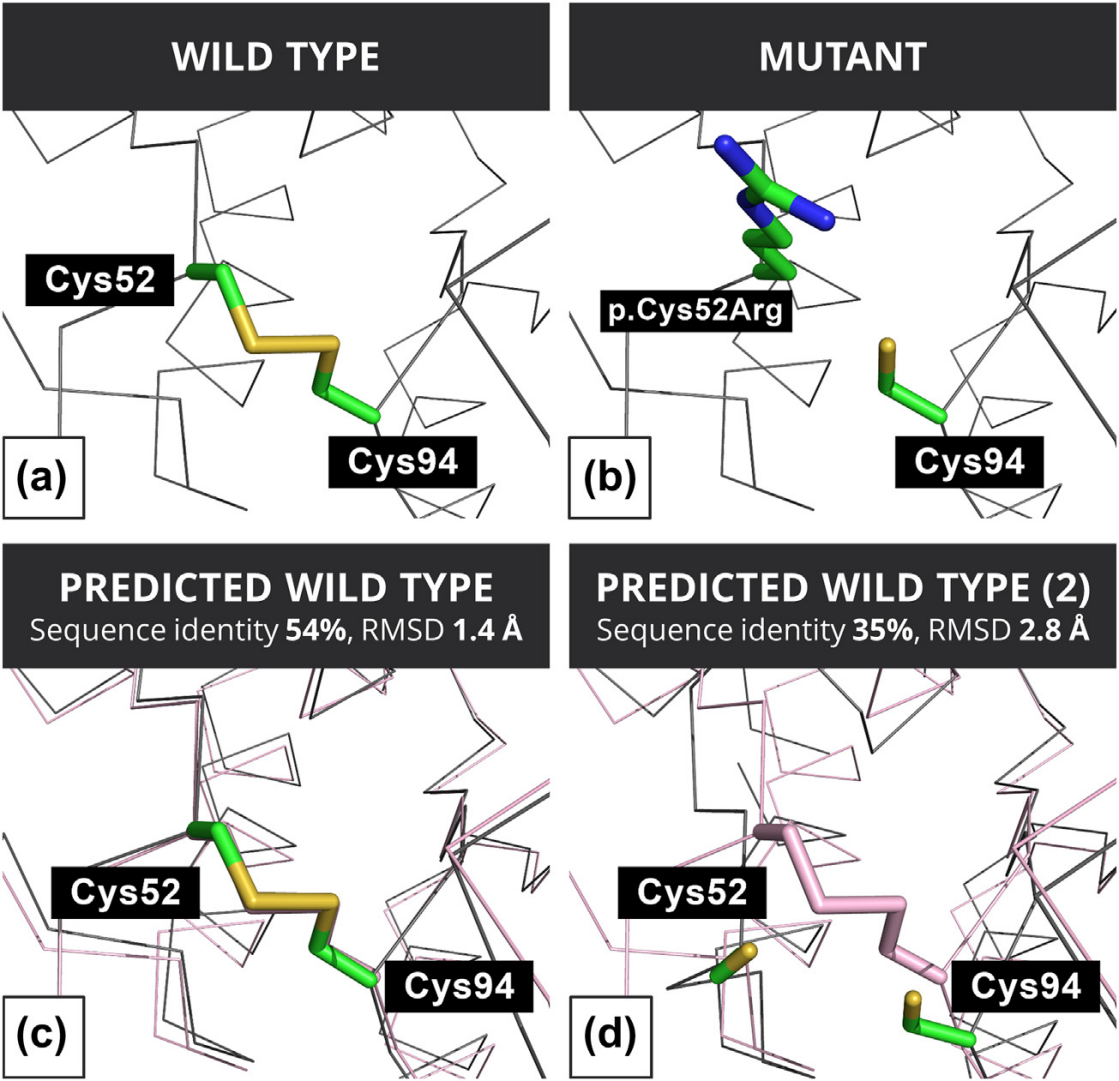
Structural analysis of variant p.Cys52Arg in the crystal and predicted structures of alpha-galactosidase. (a) Cys 52 in the wild type, (b) Arg 52 in the mutant modeled from the wild type, (c) Phyre2 wild-type predicted structure of the Cys 52 using a model with RMSD to the crystal structure of 1.4 Å (PREDICTED WILD TYPE) and (d) Phyre2-predicted wild-type structure of the Cys 52 using a model with RMSD to the crystal structure of 2.8 Å (PREDICTED WILD TYPE (2)). Color scheme for the four panels as in Fig. 7. In addition, the SG atoms are shown in yellow.

Carbonic anhydrase II (UniProt ID: P00918), code by *CA2*, is involved in physiological processes associated with CO_2_. The pathogenic missense variant p.His107Tyr destabilizes the folding of the protein [53]. This and other variants in this gene are associated with osteopetrosis with renal tubular acidosis (MIM 259730) [53], a rare condition causing severe mental and physical impairment. In the 1.1-Å wild-type structure (PDB ID: 2FOS), there are two salt bridges between His 107 and Glu 117 with N–O distances of 2.6 and 3.8 Å (Fig. 6a). The variant p.His107Tyr results in the abolition of these salt bridges (Fig. 6b). The Phyre2-predicted structure had 36% sequence identity to the template (PDB ID: 1JD0) and resulted in a model with RMSD to the experimental structure of 2.6 Å. In the predicted structure, the His–Glu salt bridge remains identified with the N–O distances being 2.8 and 3.9 Å (Fig. 6c). Thus, despite the predicted model having a low percent identity to the template, the predicted structure remained sufficiently accurate in this region to identify that there is a salt bridge in the wild type, which is not present in the mutant structure (Fig. 6d).Alpha-galactosidase (UniProt ID: P06280), coded by *GLA*, is an enzyme responsible for hydrolysing terminal, non-reducing alpha-d-galactose residues in alpha-d-galactosides. Mutations in *GLA* cause Fabry disease (MIM:301500), a rare genetic condition [55]. The mutation p.Cys52Arg causes the loss of expression and activity of the protein [55]. In our structural analysis based on the 1.9-Å resolution PDB structure 3HG3, Cys 52 (corresponding to Cys52 in PBD ID: 3HG3) forms a disulfide bond with Cys94 with an S–S bond distance of 2.08 Å (Fig. 7a). The modeling of the substitution of Cys 52 by Arg then results in breaking the disulfide bond (Fig. 7b). Our more-accurately Phyre2-predicted wild-type structure has 54% identity to the template (PDB ID: 1KTB) and has an RMSD with the experimental structure of 1.4 Å. The wild-type disulfide remains identified with an S–S distance of 2.00 Å (Fig. 7c), and therefore, the Cys to Arg substitution is correctly identified as structurally damaging in the mutant structure. In contrast, in the less-accurately Phyre2-predicted wild-type structure that is based on a model with 35% identity to the template (PDB ID: 4NZJ) and has an RMSD of 2.86 Å to the experimental structure, the wild-type disulfide bond is not identified as the S–S distance is 4.0 Å (Fig. 7d) and so the variant is not considered as introducing a damaging change into the structure.

### Missense3D web server

We have made our *in-house* pipeline for the structural analysis of missense variants, Missense3D, freely available to the scientific community as a web-server at http://www.sbg.bio.ic.ac.uk/~missense3d. There are two possible inputs:

Input A (position on protein sequence): the user provides the UniProt ID of the query protein, amino acid position on the protein sequence, wild-type residue and substitution and specifies the PDB coordinates file and chain identifier in the coordinates file (3D coordinates files cannot be uploaded in this Input). Missense 3D automatically generates the UniProt to PDB residue mapping;

Input B (position on 3D structure): the user needs to upload a 3D coordinate file, either by specifying a PDB code or by providing their own coordinate set. Next, the user specifies the amino acid position on the 3D structure (there is a warning reminding that the residue position on the PDB coordinates file may not necessarily correspond to the residue position on the amino acid sequence), wild-type residue and substitution and chain identifiers in the 3D coordinates file. Linking protein sequence and 3D coordinates is a reoccurring problem and, as Missense3D will accept predicted models from any server, we cannot guarantee a correct mapping of a residue with a sequence-based numbering onto the coordinates.

A link to our Phyre2 [56] model prediction server is also provided to aid users not familiar with generating homology modeling. The output from Missense3D is a report of the changes in structural features introduced by the amino acid substitution that are predicted to be structurally damaging. The 3D coordinate file giving the conformation of the modeled mutant structure is also provided. The result is typically returned in about 3 min. Many protein structure prediction servers, including Phyre2 [56], predict the global conformation of the protein and are not designed to model the structural consequences of a particular missense variant. Thus, Missense3D has a valuable role in augmenting the information provided by many protein structure prediction servers.

## Discussion

Bhattacharya *et al*. [30] studied 374 missense variants in 334 PDB structures. They found that 44.5% of the disease-associated substitutions and 3.4% of the neutral substitutions affected protein stability. Our analysis identified structural alerts in 40.1% of disease-associated substitutions, and our FPR was 11.5%. Thus, our TPR is very close to that of Bhattacharya *et al*., and the FPRs are in broad agreement. In contrast, SAAPdap [36] modeled the structural effects of missense variants and identified 63.2% disease-associated and 30.0% neutral missense variants as affecting protein stability. These rates are higher than ours and that of Bhattacharya *et al*., particularly with regard to overpredicting that neutral substitutions are structurally damaging. However, different data sets have been used in these three studies and this hinders direct comparisons. In addition, in SAAPdap, the structural alerts by themselves are not used as the final predictor. Al-Numair and Martin [36] used a random forest to combine the structural features with a consideration of sequence conservation and reported a cross-validated prediction accuracy (number of correct predictions of both neutral and disease/total number of variants) of 84.6% on their data set, outperforming SIFT and PolyPhen-2, which gave accuracies of 69.0% and 78.5%. Unfortunately, because SAAPdap does not allow uploading 3D coordinates files and does not allow performing a batch analysis, we could not use it to compare our results. The SDM2 server, which allows uploading 3D coordinates files, does not return a detailed structural analysis, such as the one performed in this study, thus again preventing comparison with our results.

The challenges in robust evaluation of the accuracies of algorithms to predict the phenotypic effect of variants have been highlighted [23], [57]. Some proteins, often those that are essential, have many missense variants that are disease-associated, other proteins are found to have many missense variants that do not markedly alter the phenotype, and others have a similar number of disease-associated and neutral variants. Reported predictive accuracies, such as the TPR and FPR, are thus greatly affected by the data set of proteins used in the study, and caution must be used in comparing accuracy measures from different groups. The values reported in this study should therefore be considered as approximate guidelines.

At present, our pipeline, Missense3D, available as a website, should be used to provide a qualitative description of potential structurally damaging missense variants in both experimental and homology-predicted structures. Further work could use a cross-validated machine learning approach to extend these structural alerts to yield a quantitative predictor. In this version of Missense3D, we do not take into account disruption of protein–protein, protein–DNA and protein–small ligand interactions, which could greatly enhance the accuracy of Missense3D. For this reason, MCC may not be the best metric to assess the value of Missense3D.

One limitation of our work is that we did not compare the results obtained from Phyre2 models with those that could be obtained by using other modeling servers, such as I-Tasser [58] or Rosetta [59]. We acknowledge that a model generated by different servers could yield slightly different results. It is possible that using a predictor, such as I-Tasser, which consistently performed exceptionally well at CASP [60], could provide marginally better results. However, the conclusion of the paper is that the Missense3D is robust when applied to models. Another limitation of our study is that, ideally, we would have wished to compare the results of Missense3D to similar existing algorithms. However, technical reasons, such as the lack of batch facilities or ability to upload your own structures, prevented such an analysis.

In conclusion, overall, in terms of the TPR/FPR ratio, the structural analysis of missense variants is nearly as good in predicted structures, even those with low-sequence identity to the template, as can be obtained analyzing experimental structures. Since jointly experimental structures and predicted homology models cover over 50% of residues in the human proteome [12], the effect of many human missense variants can be interpreted from a structural perspective. Our novel method Missense3D is a useful tool for assessing whether a missense variant is likely to have a damaging effect on the stability of the folded protein, and it is applicable to both experimentally determined structures and homology-predicted models.

## Methods

### Data set of high-quality experimental structures

Human high-quality x-ray structures (resolution < 2.0 Å) were obtained from the representative MolProbity's Top8000 database [37]. Nine hundred ninety-nine structures were obtained, which could be mapped unambiguously to a UniProt ID [40].

The structural analysis was repeated on a second independent data set of 855 structures with lower resolution (< 2.5 Å, extracted from PDB) and corresponding to 565 human proteins. These proteins were being used for our subsequent analysis on quaternary structures (not reported) and were not identified until all features and the method of analysis were established on the MolProbity and the Phyre2-predicted structures.

### Data set of missense variants

Missense variants were curated from Humsavar (from UniProt [61] release 4 Feb 2015), ClinVar [39] (release 7 Jan 2015) and ExAC [40] (version 0.3, release 13 Jan 2015) (see Ref. [62]).

Variants were classified as disease associated if reported as pathogenic in ClinVar or disease causing in UniProt. Variants were classified as neutral if they were reported as “benign” in ClinVar or “polymorphisms” in UniProt (and no association with disease was present in ClinVar). Variants reported in the ExAC database were included in the neutral set if no association with disease was reported in ClinVar or UniProt.

The initial data consist of 26,884 disease-associated and 563,099 neutral variants. Of these, 1965 disease-associated and 2134 neutral variants could be mapped onto 606 of the 999 PDB structures. The neutral variants were also divided according to their MAF into 1550 rare variants (MAF < 0.01), 273 common variants (MAF ≥ 0.01) and 311 variants of unknown frequency (no MAF reported). A second set of neutral (2518) and disease-causing (3718) variants from Humsavar was mapped onto the lower-resolution data set of 855 PDB structures. Sequence to structure mapping was performed by aligning UniProt amino acid sequence to PDB amino acid sequence using ClustalW [63].

### Data set of predicted structures

Phyre2 [56] in normal mode was used to predict the structures of the 606 proteins from their sequences. The resultant models were filtered to obtain models that meet the following criteria: (i) sequence identity > 30% but ≤ 95% (the upper bound was set to avoid the bias of the same or a very similar PDB as in Top8000 being used as the template for homology modeling); (ii) the PDB code of the template was different from that of the corresponding experimental structure; and (iii) confidence that the template is correct is ≥ 90%. This score ranges from 0 to 100 and represents the probability that the match between the query sequence and the template is a true homology. A Phyre2 [56] confidence > 90% suggests that the core of the protein is modeled at high accuracy (2–4 Å RMSD from the native, true structure]; (iv) the residues in the model covered ≥ 80% of the corresponding experimental structure; and (v) the model length was > 50 residues. Models for 471 of these 606 proteins were obtained. Phyre2 can generate up to 20 models using different templates and at different sequence identities. One thousand fifty-two Phyre2 models for the 471 proteins were generated and then separated into seven bins according to their sequence identity to the template (30%–39%, … 80%–89% and 90%–95%). If there were several models in a bin, we selected one representative taking first the highest-resolution template, then an NMR template and finally a template with unspecified resolution.

### Calculation of damaging structural features

We used widely accepted distance cut offs for disulfide bonds [64], hydrogen bonds [65] and salt bridges [66] to which we added 1 Å to allow for errors in the modeling of the mutant by SCWRL4 [41] and in the prediction of the coordinates of the model. A residue is identified as buried if its relative solvent accessibility [48] was less than 9%. Hydrophobic residues are as follows: A, C, F, I, L, M, V and W; hydrophilic residues are as follows: D, E, H, K, N, Q and R, with the others being neutral (G, P, S, T and Y). D and E are treated as negatively charged and H, K and R as positively charged. We note that there are several variations to these definitions of residue properties.

For the three structural features calculated using a distance (disulfide bonds, hydrogen bonds and salt bridges), a control was introduced. A structure (WT-CONTROL) was generated using SCWRL4 allowing every side chain (including the wild-type residue at the variant position) that was adjusted in MUTANT to be repacked. WT and WT-CONTROL were compared for hydrogen bonds, salt bridges and disulfide bonds. If any of these were different between WT and WT-CONTROL, then that feature was excluded from the comparison of WT and MUTANT.

In order to validate our results, we also used FoldX [18] to generate the mutant structures.

*Disulfide bond breakage*: A substitution was considered damaging when the wild type had a disulfide bond, which is disrupted in the mutant structure. A disulfide bond is defined as a bond between two sulfur atoms of Cys residues that are < 3.3 Å apart.

*Buried Pro introduced*: Substitutions in the core of a protein tend to be particularly damaging. The introduction of a proline with its restricted backbone conformation is potentially deleterious to maintaining the wild-type protein structure.

*Clash*: We used “clashlistcluster” from MolProbity [37]. In MolProbity, a clash score of over 30 indicates a poor structure. Since we are focusing on the effect of a variant rather than the entire structure, the clash score is measured locally considering only atoms within 20 Å from the Cα of the variant residue. Here a substitution is regarded as damaging if the local MolProbity clash score is over 30 and the increase in local clash when compared to the local clash score of the wild-type structure is ≥ 18.

*Buried hydrophilic introduced*: A substitution is considered damaging when the wild-type residue is buried and hydrophobic and the substitution is a hydrophilic residue.

*Buried charge introduced*: A substitution is regarded as damaging when the wild-type residue is buried and is not a charged residue and the substitution is a charged residue.

*Buried charge switch*: A substitution is considered damaging if the wild-type residue is buried and charged and the substitution introduces a residue with an opposite charge.

*Secondary structure altered*: Secondary structure is defined as one of eight classes by DSSP [67]: H, α-helix; B, β-bridge; E, strand; G, helix-3; I, helix-5; T, turn; and S, bend (sharp turn); and C, coil. A substitution is considered damaging if it results in a change in the DSSP secondary structure assignment.

*Buried charge replaced*: A substitution is considered damaging if the original residue is buried and charged and the substitution introduces an uncharged residue. Buried charged residues typically form electrostatic interaction with a nearby residue of opposite charge. Substituting a charged residue with an uncharged one can affect protein stability.

*Disallowed phi/psi*: A substitution is considered damaging if the variant in the MUTANT structure is predicted to be outside the allowed phi/psi regions, whereas the wild-type residue is identified as in a favored or allowed region. Torsion angles were calculated using ‘Ramachandran’ from MolProbity [37].

*Buried Gly replaced*: Buried glycines are normally highly conserved. A substitution that replaces a buried glycine with any other residue is therefore considered damaging.

*Buried H-bond breakage*: A substitution is considered damaging if it disrupts all side-chain hydrogen bonds (both side-chain to main-chain and side-chain to side-chain) that were formed by the wild-type residue. Missense3D considers separately the effect on main-chain/main-chain hydrogen bonding via alteration in the assigned secondary structure. H-bonds are defined by a N–O distance of < 3.9 Å.

*Buried salt bridge breakage*: A substitution is considered damaging when the wild-type residue is buried, and it forms a salt bridge with another residue and the substitution no longer forms a salt bridge. Salt bridges between oppositely charge side chains are identified by an N–O distance of < 5 Å.

*Cavity altered*: Changes in cavity volume can affect the protein stability. Cavity volumes on both the wild-type (WT) and mutant (MUTANT) structures are measured using KVFinder [68]. The sizes of the probes were set to their default values: 1.4 Å for probe in and 4.0 Å for probe out. We consider a substitution as damaging if it results in increase or decrease of a cavity volume of at least 70 Å^3^. This is consistent with an upper limit of most observed cavities in proteins [69].

*Buried*/*exposed switch*: A substitution is considered damaging if it results in a switch between the buried and exposed states of the wild-type and mutant residues.

*Cis Pro replaced*: A substitution from proline which originally was in a cis-conformation is considered as damaging. Although the omega angle is theoretically close to 0°, this value may vary, particularly in predicted models. Thus, we allow a − 45° to 45° range for the omega angle to be considered in the cis conformation.

*Gly in a bend*: Glycine, with only hydrogen as its side chain, can adopt a far larger region in the phi/psi backbone dihedral angle space than other side chains. Glycine is found in loops and regions where a polypeptide chain makes a sharp turn. Accordingly, glycine is often conserved. We consider a substitution to any amino acid as damaging if the wild-type residue is glycine and its DSSP-assigned secondary structure is “S” for bend or sharp turn.

*Exposed hydrophobic introduced (evaluated but not used in the final pipeline)*: A substitution is considered damaging when the wild-type residue is exposed and hydrophilic and the substitution is hydrophobic.

The following features were considered to be solely dependent on the wild-type structure:

Disulfide bond breakage, Buried Pro introduced, Buried hydrophilic introduced, Buried charge introduced, Buried charge switch, Disallowed phi/psi, Buried charge replaced, Buried Gly replaced, Cis Pro replaced and Gly in a bend. The remaining features were considered dependent on the model quality.

### Statistical evaluation of performance

A disease-associated substitution that is identified as having at least one structurally damaging feature is regarded as a TP. A neutral substitution reported to have at least one structural problem is regarded as a FP. We denote the total numbers of positives (i.e., disease-associated variants) and negatives (i.e., neutral variants) as NP and NN, respectively. Performance was evaluated using TPR, FPR and their ratio: TPR = TP/ NP, FPR = FP/ NN and Ratio = TPR/FPR. Sensitivity and specificity are related to TPR and FPR: sensitivity = TPR, specificity = (1-FPR).

A one-tailed test of the difference of two unpaired proportions [70] was used to assess whether the TPR for a particular structural alerts in all disease-associated variants (NP = 1965) was significantly greater than the FPR in all neutral variants (NN = 2134). These significance values were correct using Benjamini–Hochberg for multiple testing [42].

95% confidence intervals (CI) were assigned to values for TPR and FPR:

$\mathrm{TPR}\pm 1.96\sqrt{\frac{\left(\mathrm{TPR}\right)\left(1-\mathrm{TPR}\right)}{\mathrm{NP}}}$ and $\mathrm{FPR}\pm 1.96\sqrt{\frac{\left(\mathrm{FPR}\right)\left(1-\mathrm{FPR}\right)}{\mathrm{NN}.}}$

The MCC was used to account for imbalance in the numbers of disease-associated and neutral variants.

$\mathrm{MCC}=\frac{\left({\mathrm{TP}}^{*}\mathrm{TN}\right)-\left({\mathrm{FP}}^{*}\mathrm{FN}\right)}{{\sqrt{\left({\mathrm{TP}}^{*}\mathrm{FP}\right)}}^{*}\left({\mathrm{TP}}^{*}\mathrm{FN}\right)*\left({\mathrm{TN}}^{*}\mathrm{FP}\right)*\left({\mathrm{TN}}^{*}\mathrm{FN}\right)}$

### Accession numbers

UniProt ID:Q9H2X3, UniProt ID:P02749, UniProt ID: P00918, UniProt ID: P06280, UniProt ID: P16581.

PDB ID:1XPH, PDB ID:3OP8, PDB ID:1G1T, PDB ID: 2FOS, PDB ID: 1JD0, PBD ID: 3HG3, PDB ID: 1KTB, PDB ID: 4NZJ.

### Data availability

The data that support the findings of this study and the data generated in this study are available from the data set page of our Missense3D website at http://www.sbg.bio.ic.ac.uk/~missense3d/.

## Appendix A Supplementary data

Supplementary material Image 1
