# Supplementary material for: Can Predicted Protein 3D Structures Provide Reliable Insights into whether Missense Variants Are Disease Associated?
Source: J Mol Biol. 2019 May 17;431(11):2197–212. doi: 10.1016/j.jmb.2019.04.009 (PMC6544567; doi:10.1016/j.jmb.2019.04.009)

**Supplementary Material**

**Can Predicted Protein 3D-structures provide Reliable Insights into whether Missense Variants are Disease-associated?**

**Sirawit Ittisoponpisan, Suhail A. Islam, Tarun Khanna, Eman Alhuzimi, Alessia David (*) and Michael J. E. Sternberg (*)**

(*) Joint corresponding authors

**Table S1 – Structural analysis on experimental structures**

| **Feature assessed as a structural impact** | **TP, n** | **FP, n** | **TPR**± **CI** | **FPR**± **CI** | **P-Value**  **(1-tailed)** |
| --- | --- | --- | --- | --- | --- |
| Disulphide bond breakage | 70 | 3 | 3.56 ± 0.82 | 0.14 ± 0.16 | < 0.0001 |
| Buried Pro introduced | 64 | 8 | 3.26 ± 0.78 | 0.37 ± 0.26 | < 0.0001 |
| Clash | 103 | 13 | 5.24 ± 0.99 | 0.61 ± 0.33 | < 0.0001 |
| Buried hydrophilic introduced | 81 | 11 | 4.12 ± 0.88 | 0.52 ± 0.30 | < 0.0001 |
| Buried charge introduced | 170 | 26 | 8.65 ± 1.24 | 1.22 ± 0.47 | < 0.0001 |
| Buried charge switch | 19 | 4 | 0.97 ± 0.43 | 0.19 ± 0.18 | 0.0004 |
| Secondary structure altered | 21 | 4 | 1.07 ± 0.45 | 0.19 ± 0.18 | 0.0001 |
| Buried charge replaced | 99 | 25 | 5.04 ± 0.97 | 1.17 ± 0.46 | < 0.0001 |
| Disallowed phi/psi | 115 | 29 | 5.85 ± 1.04 | 1.36 ± 0.49 | < 0.0001 |
| Buried Gly replaced | 115 | 33 | 5.85 ± 1.04 | 1.55 ± 0.52 | < 0.0001 |
| Buried H-bond breakage | 148 | 53 | 7.53 ± 1.17 | 2.48 ± 0.66 | < 0.0001 |
| Buried salt bridge breakage | 62 | 23 | 3.16 ± 0.77 | 1.08 ± 0.44 | < 0.0001 |
| Cavity altered | 105 | 41 | 5.34 ± 0.99 | 1.92 ± 0.58 | < 0.0001 |
| Buried / exposed switch | 83 | 38 | 4.22 ± 0.89 | 1.78 ± 0.56 | < 0.0001 |
| Cis Pro replaced | 7 | 4 | 0.36 ± 0.26 | 0.19 ± 0.18 | 0.1483 |
| Gly in a bend | 43 | 25 | 2.19 ± 0.65 | 1.17 ± 0.46 | 0.0054 |
| Exposed hydrophobic introduced ** | 103 | 177 | 5.24 ± 0.99 | 8.29 ± 1.17 | 0.9999 |
|  |  |  |  |  |  |

TP: true positive assignments; FP: false positive assignments; TPR ± CI: TP rate (= TP/ NP, where NP = 1,956) ± 95% confidence interval; FPR ± CI, FP rate (=FP/NN, where NN = 2,134) ± 95% confidence interval; P-value: one-tailed difference of two proportions.

**, this feature was evaluated but not used.

**Table S2 – Comparison of results obtained on experimental structures by using default or relaxed cut-off distances**.

| **Feature** | **TP** | **FP** | **TPR** | **FPR** | **TPR/FPR** |
| --- | --- | --- | --- | --- | --- |
| *Disulphide bond breakage (2.3Å)* | 65 | 3 | 3.31 | 0.14 | 23.53 |
| *Disulphide bond breakage (3.3Å)* | 70 | 3 | 3.56 | 0.14 | 25.34 |
| *Buried salt bridge breakage (4.0Å)* | 13 | 5 | 0.66 | 0.23 | 2.82 |
| *Buried salt bridge breakage (5.0Å)* | 62 | 23 | 3.16 | 1.08 | 2.93 |
| *Buried H bond breakage (2.9Å)* | 197 | 81 | 10.03 | 3.8 | 2.64 |
| *Buried H bond breakage (3.9Å)* | 148 | 53 | 7.53 | 2.48 | 3.03 |

**Table S3 – Predictions obtained using SCWRL4-generated mutant structures**

The True Positive Rate (TPR) is 40.1%, False Positive Rate (FPR) is 11.4% and TPR/FPR = 3.51

|  |  | **Observed** | |
| --- | --- | --- | --- |
|  |  | **Neutral** | **Damaging** |
| **Predicted** | **Neutral** | 1890 | 1179 |
|  | **Damaging** | 244 | 786 |

**Table S4 – Predictions obtained using FoldX-generated mutant structures**

The True Positive Rate (TPR) is 40.0%, False Positive Rate (FPR) is 16.0%; TPR/FPR = 2.5

|  |  | **Observed** | |
| --- | --- | --- | --- |
|  |  | **Neutral** | **Damaging** |
| **Predicted** | **Neutral** | 1793 | 1179 |
|  | **Damaging** | 341 | 786 |

**Table S5 – Comparison of the results obtained using SCWRL-generated versus FoldX-generated mutant structures.**

|  |  | **SCWRL4-generated** | |
| --- | --- | --- | --- |
|  |  | **Incorrect** | **Correct** |
| **FoldX-generated** | **Incorrect** | 1295 | 225 |
|  | **Correct** | 128 | 2451 |

p<0.001, McNemar test **Table S6 – Comparison, at feature level, of the predictions made using SCWRL4-generated and FoldX-generated mutant structures**

| **SCWRL4** | | | | **FoldX** | | |
| --- | --- | --- | --- | --- | --- | --- |
|  | TPR (%) | FPR (%) | TPR/FPR (%) | TPR (%) | FPR (%) | TPR/FPR (%) |
| Overall | 40.1 | 11.4 | 3.5 | 40.0 | 16.0 | 2.5 |
| Disulphide bond breakage | 3.6 | 0.1 | 25.3 | 3.6 | 0.1 | 25.3 |
| Buried Pro introduced | 3.3 | 0.4 | 8.7 | 3.2 | 0.4 | 8.5 |
| Clash | 5.2 | 0.6 | 8.6 | 0.7 | 0.5 | 1.5 |
| Buried hydrophilic introduced | 4.4 | 0.5 | 8.5 | 4.3 | 0.5 | 9.1 |
| Buried charge introduced | 8.9 | 1.2 | 7.1 | 8.4 | 0.9 | 9.3 |
| Buried charge switch | 1.0 | 0.2 | 5.2 | 0.8 | 0.2 | 4.3 |
| Secondary structure altered | 1.1 | 0.2 | 5.7 | 1.1 | 0.2 | 5.7 |
| Buried charge replaced | 5.0 | 1.2 | 4.3 | 5.0 | 1.4 | 3.5 |
| Disallowed phi/psi | 5.9 | 1.4 | 4.3 | 5.9 | 1.4 | 4.3 |
| Buried Gly replaced | 5.9 | 1.6 | 3.8 | 5.8 | 1.6 | 3.6 |
| Buried H-bond breakage | 7.5 | 2.5 | 3.0 | 5.7 | 2.3 | 2.5 |
| Buried salt bridge breakage | 3.2 | 1.1 | 2.9 | 2.9 | 0.9 | 3.1 |
| Cavity altered | 5.3 | 1.9 | 2.8 | 7.9 | 7.8 | 1.0 |
| Buried / exposed switch | 4.3 | 1.8 | 2.4 | 4.6 | 2.3 | 2.0 |
| Cis Pro replaced | 0.4 | 0.2 | 1.9 | 0.4 | 0.2 | 1.9 |
| Gly in a bend | 2.2 | 1.2 | 1.9 | 2.2 | 1.2 | 1.9 |

Highlighted in grey are wild-type dependent features.

TPR, True Positive Rate; FPR, False Positive Rate

**Table S7 – Structural analysis on an independent dataset of 855 structures with lower resolution (<2.5 Å)**

| **Feature assessed as a structural impact** | **True positives (TP), n.** | **False positives (FP), n.** | **TPR**± **CI** | **FPR**± **CI** | **P-Value**  **(1-tailed)** |
| --- | --- | --- | --- | --- | --- |
| ***Disulphide bond breakage*** | 118 | 12 | 3.17 ± 0.56 | 0.48 ± 0.27 | < 0.0001 |
| ***Buried Pro introduced*** | 158 | 27 | 4.25 ± 0.65 | 1.07 ± 0.40 | < 0.0001 |
| ***Clash*** | 178 | 26 | 4.79 ± 0.69 | 1.03 ± 0.39 | < 0.0001 |
| ***Buried hydrophilic introduced*** | 149 | 31 | 4.01 ± 0.63 | 1.23 ± 0.43 | < 0.0001 |
| ***Buried charge introduced*** | 328 | 64 | 8.82 ± 0.91 | 2.54 ± 0.61 | < 0.0001 |
| ***Buried charge switch*** | 33 | 7 | 0.89 ± 0.30 | 0.28 ± 0.21 | 0.0016 |
| ***Secondary structure altered*** | 53 | 10 | 1.43 ± 0.38 | 0.40 ± 0.25 | 0.0001 |
| ***Buried charge replaced*** | 151 | 26 | 4.06 ± 0.63 | 1.03 ± 0.39 | < 0.0001 |
| ***Disallowed phi/psi*** | 194 | 35 | 5.22 ± 0.71 | 1.39 ± 0.46 | < 0.0001 |
| ***Buried Gly replaced*** | 193 | 41 | 5.19 ± 0.71 | 1.63 ± 0.49 | < 0.0001 |
| ***Buried H-bond breakage*** | 225 | 74 | 6.05 ± 0.77 | 2.94 ± 0.66 | < 0.0001 |
| ***Buried salt bridge breakage*** | 102 | 19 | 2.74 ± 0.52 | 0.75 ± 0.34 | < 0.0001 |
| ***Cavity altered*** | 202 | 96 | 5.43 ± 0.73 | 3.81 ± 0.75 | 0.0019 |
| ***Buried / exposed switch*** | 141 | 48 | 3.79 ± 0.61 | 1.91 ± 0.53 | < 0.0001 |
| ***Cis Pro replaced*** | 15 | 5 | 0.40 ± 0.20 | 0.20 ± 0.17 | 0.0848 |
| ***Gly in a bend*** | 60 | 22 | 1.61 ± 0.40 | 0.87 ± 0.36 | 0.0059 |

TP: true positive assignments; FP: false positive assignments; TPR ± CI: TP rate (= TP/ NP, where NP = 3,718) ± 95% confidence interval; FPR ± CI, FP rate (=FP/NN, where NN = 2,516) ± 95% confidence interval; P-value: one-tailed difference of two proportions.

**Table S8 – Structural analysis on predicted structures**

| **Sequence identity** |  | **TPR (± 95% CI)** | **FPR (± 95% CI)** |
| --- | --- | --- | --- |
| ***30-39*** | MODEL | 34.75 ± 2.62 | 11.25 ± 1.76 |
|  | EXP | 36.08 ± 2.64 | 11.58 ± 1.78 |
| ***40-49*** | MODEL | 36.01 ± 3.02 | 12.53 ± 2.16 |
|  | EXP | 40.12 ± 3.08 | 12.42 ± 2.15 |
| ***50-59*** | MODEL | 35.88 ± 3.08 | 12.64 ± 2.59 |
|  | EXP | 35.55 ± 3.07 | 11.85 ± 2.52 |
| ***60-69*** | MODEL | 37.21 ± 4.04 | 13.07 ± 3.52 |
|  | EXP | 35.75 ± 4.00 | 10.80 ± 3.24 |
| ***70-79*** | MODEL | 24.77 ± 8.10 | 11.35 ± 4.57 |
|  | EXP | 20.18 ± 7.54 | 11.89 ± 4.66 |
| ***80-89*** | MODEL | 26.62 ± 5.34 | 11.86 ± 4.76 |
|  | EXP | 29.28 ± 5.50 | 12.43 ± 4.86 |
| ***90-95*** | MODEL | 37.07 ± 5.88 | 11.90 ± 4.90 |
|  | EXP | 33.20 ± 5.74 | 11.31 ± 4.79 |

TP: true positive assignments; FP: false positive assignments; TPR ± CI: TP rate (= TP/ NP, where NP = 1,956) ± 95% confidence interval; FPR ± CI, FP rate (=FP/NN, where NN = 2,134) ± 95% confidence interval

**Table S9 – Prediction agreement at variant level of the results obtained from experimental structures and 3D models.**

| **Sequence Id**, **%** | **Variants,**  **n** | **Agreement** | **Disagreement** | **Features triggered to explain**  **disease-causing variants** | | |
| --- | --- | --- | --- | --- | --- | --- |
|  |  |  |  | **Complete overlap** | **Partial overlap** | **Different features** |
| 30-39 | 2516 | 2161 (86%) | 355 (14%) | 242 (58%) | 127 (31%) | 46 (11%) |
| 40-49 | 1874 | 1615 (86%) | 259 (14%) | 209 (59%) | 113 (32%) | 31 (9%) |
| 50-59 | 1564 | 1368 (87%) | 196 (13%) | 181 (58%) | 106 (34%) | 25 (8%) |
| 60-69 | 903 | 797 (88%) | 106 (12%) | 131 (69%) | 47 (25%) | 12 (6%) |
| 70-79 | 294 | 260 (88%) | 34 (12%) | 18 (62%) | 10 (35%) | 1 (3%) |
| 80-89 | 440 | 406 (92%) | 34 (8%) | 58 (74%) | 20 (26%) | 0 (0%) |
| 90-95 | 427 | 390 (91%) | 37 (9%) | 58 (63%) | 30 (33%) | 4 (4%) |

Sequence Id, sequence identity

At variant level, the comparison of predictions is labeled “Complete overlap” if all features triggered in the experimental structure are also triggered when analyzing a model, whereas the prediction is labeled “partial overlap” if there is at least one feature in common between predictions.

**Table S10 –** **Structural analysis on predicted structures using only wild type dependent features.**

Within each sequence identity bin, results are compared between models and the corresponding experimental structures.

| **Sequence**  **id (%)** | **Predicted structures** | | | **Experimental structures** | | |
| --- | --- | --- | --- | --- | --- | --- |
|  | TPR (%) | FPR (%) | TPR/FPR (%) | TPR (%) | FPR (%) | TPR/FPR (%) |
| 30-39 | 24.1 | 5.2 | 4.6 | 26.3 | 6.0 | 4.4 |
| 40-49 | 25.3 | 5.9 | 4.3 | 29.4 | 6.7 | 4.4 |
| 50-59 | 25.9 | 5.7 | 4.5 | 26.0 | 7.3 | 3.6 |
| 60-69 | 24.9 | 6.0 | 4.2 | 24.9 | 6.2 | 4.0 |
| 70-79 | 16.5 | 4.9 | 3.4 | 13.8 | 5.9 | 2.3 |
| 80-89 | 22.4 | 6.2 | 3.6 | 22.1 | 6.2 | 3.6 |
| 90-95 | 26.6 | 7.7 | 3.5 | 25.9 | 7.7 | 3.4 |

TPR, true positive rate; FPR, false positive rate

**Figure S1 – Analysis of performance of individual structural features on predicted structures.** The *prediction performance for each of the 16 structural features on predicted structures at different sequence identities is presented. For a given feature in each sequence identity bin, the fractions of positive predictions for the disease-associated and neutral variants are shown for both the predicted and the corresponding experimental structures. 95% confidence intervals on the positive rates are shown as lines.*


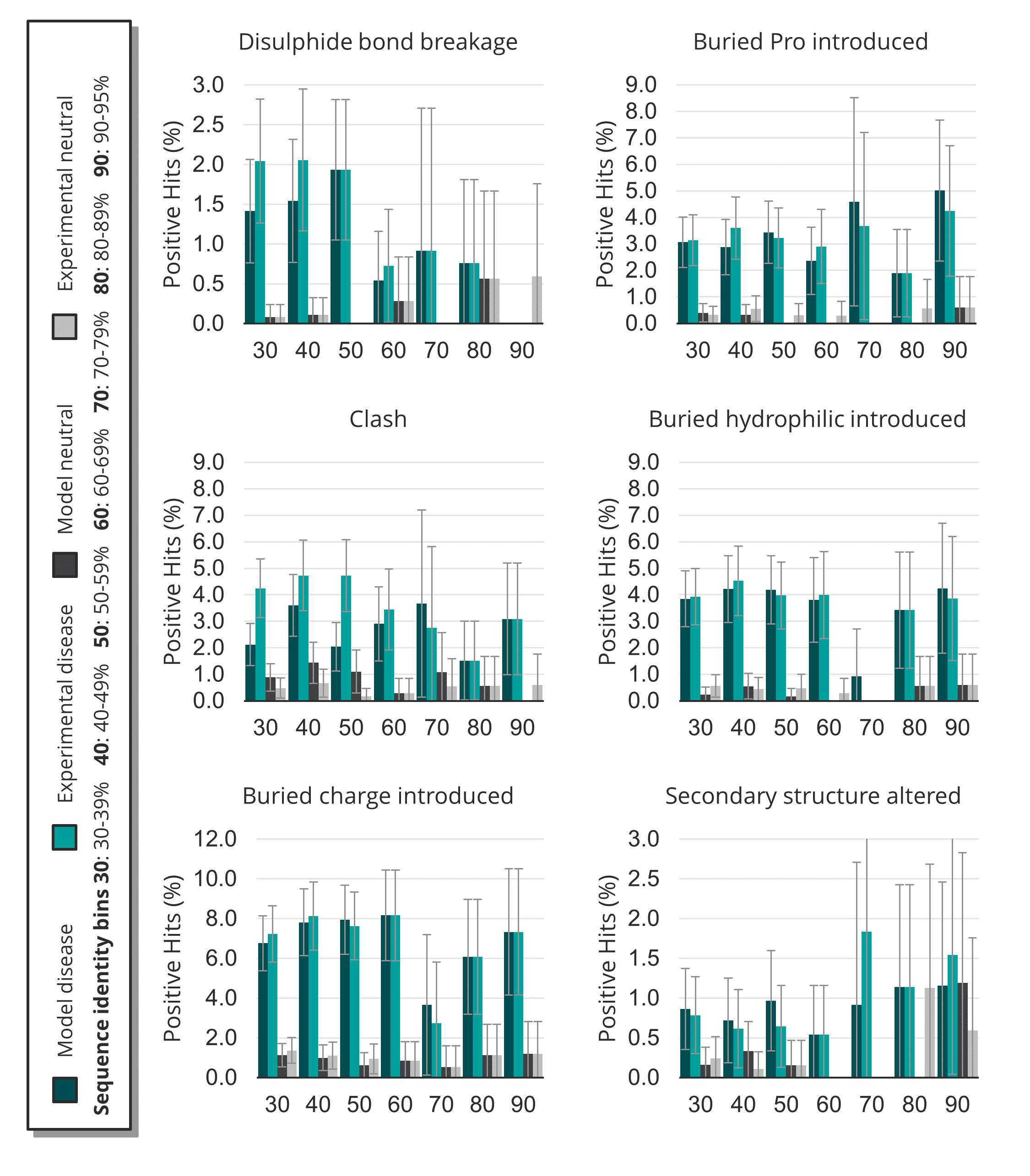


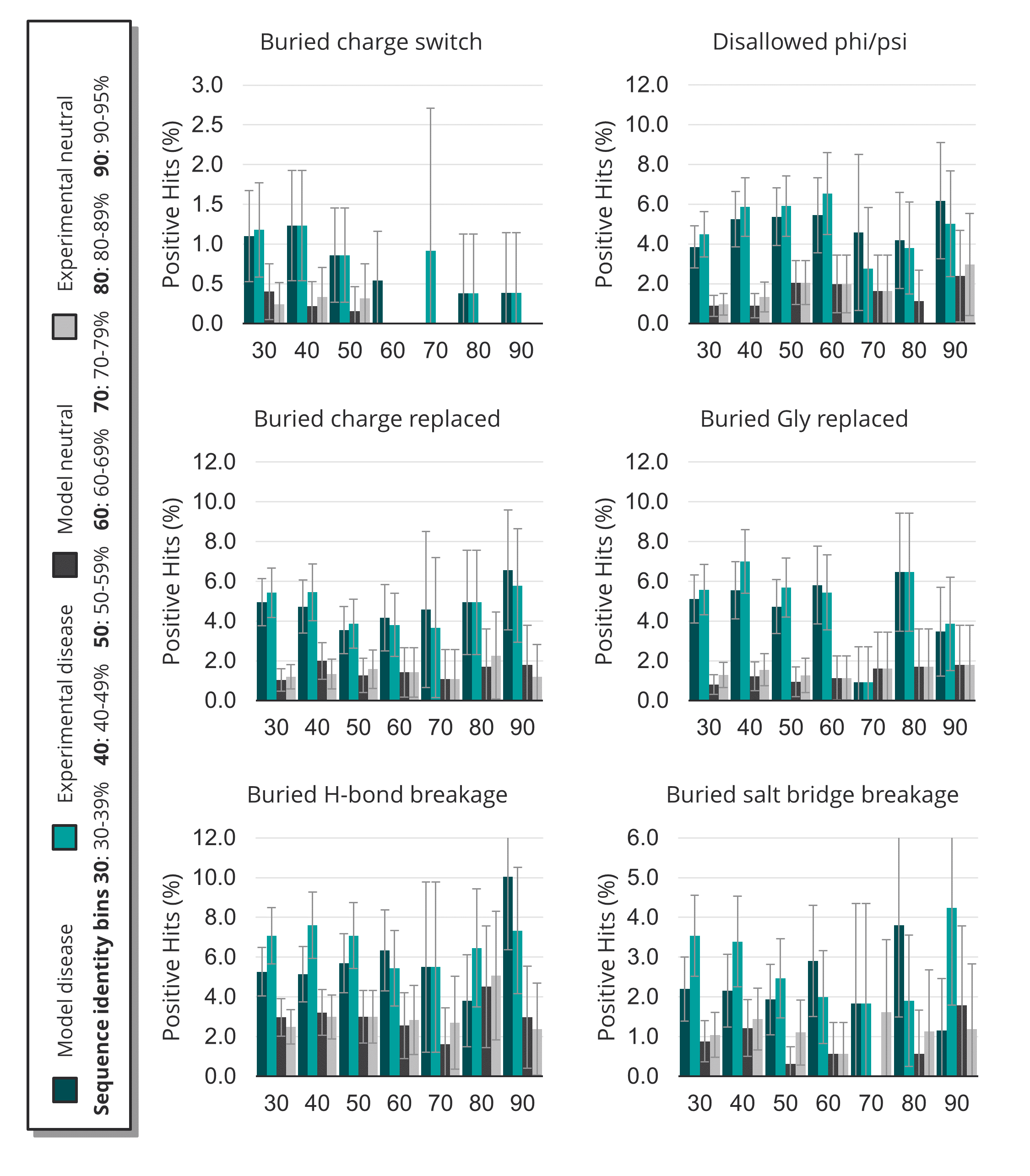


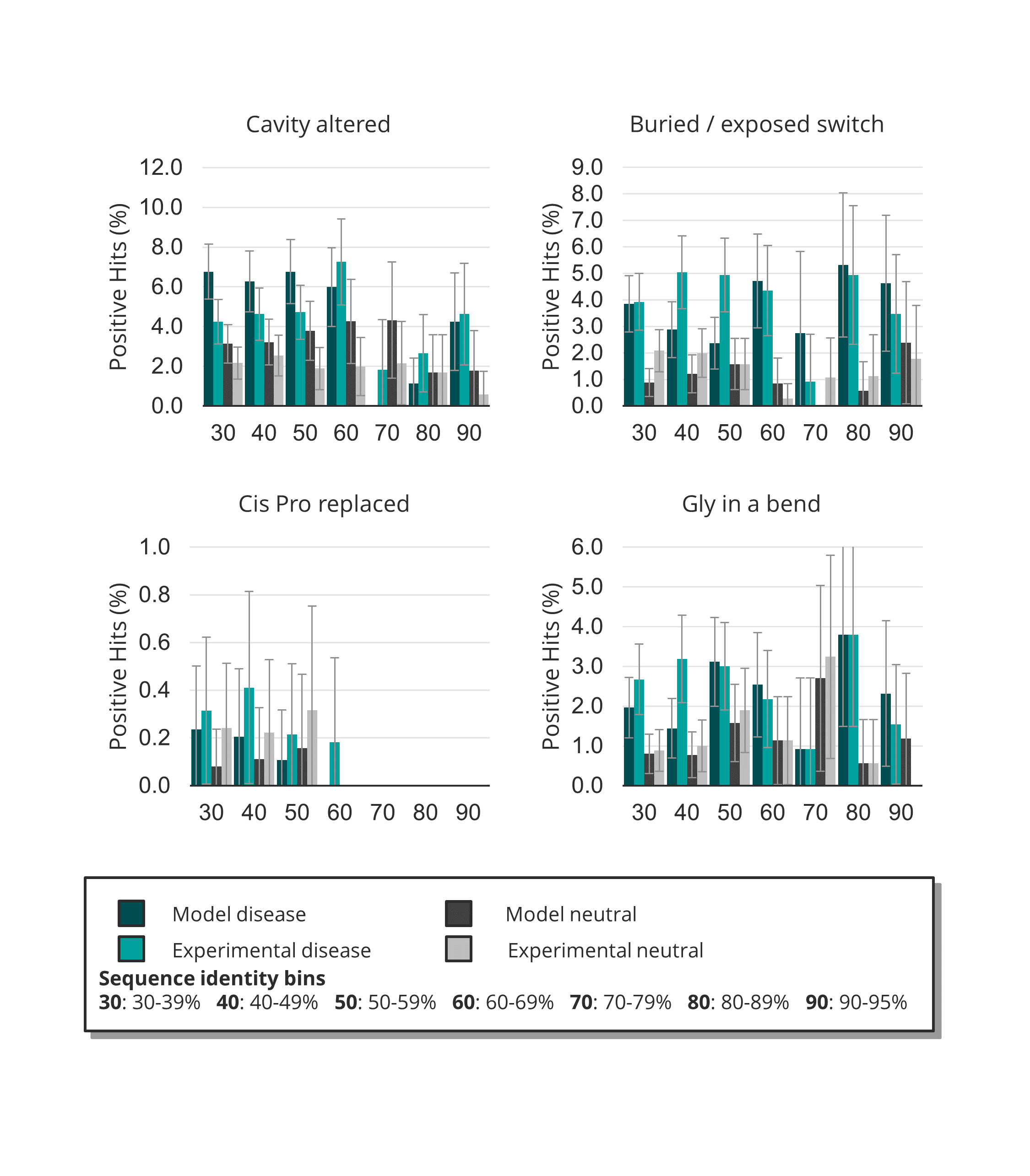

Supplement: Supplementary file 1 — Supplementary material [file mmc1.docx]
